# Involvement of D2-like dopaminergic receptors in contextual fear conditioning in female rats: influence of estrous cycle

**DOI:** 10.3389/fnbeh.2022.1033649

**Published:** 2022-11-28

**Authors:** Camila de Oliveira Alves, Adriano Edgar Reimer, Amanda Ribeiro de Oliveira

**Affiliations:** ^1^Department of Psychology, Center of Education and Human Sciences, Federal University of São Carlos (UFSCar), São Carlos, Brazil; ^2^Institute of Neuroscience and Behavior (INeC), Ribeirão Preto, Brazil

**Keywords:** conditioned fear, freezing, sulpiride, dopamine, proestrus/estrus, metestrus/diestrus, mesolimbic

## Abstract

**Introduction:** Dopamine has been increasingly recognized as a key neurotransmitter regulating fear/anxiety states. Nevertheless, the influence of sex and estrous cycle differences on the role of dopamine in fear responses needs further investigation. We aimed to evaluate the effects of sulpiride (a dopaminergic D2-like receptor antagonist) on contextual fear conditioning in females while exploring the influence of the estrous cycle.

**Methods:** First, using a contextual fear conditioning paradigm, we assessed potential differences in acquisition, expression, and extinction of the conditioned freezing response in male and female (split in proestrus/estrus and metestrus/diestrus) Wistar rats. In a second cohort, we evaluated the effects of sulpiride (20 and 40 mg/kg) on contextual conditioned fear in females during proestrus/estrus and metestrus/diestrus. Potential nonspecific effects were assessed in motor activity assays (catalepsy and open-field tests).

**Results:** No sex differences nor estrous cycle effects on freezing behavior were observed during the fear conditioning phases. Sulpiride reduced freezing expression in female rats. Moreover, females during the proestrus/estrus phases of the estrous cycle were more sensitive to the effects of sulpiride than females in metestrus/diestrus. Sulpiride did not cause motor impairments.

**Discussion:** Although no sex or estrous cycle differences were observed in basal conditioned fear expression and extinction, the estrous cycle seems to influence the effects of D2-like antagonists on contextual fear conditioning.

## Introduction

Anxiety-related disorders are complex heterogeneous mental disorders and important causes of health-related burden worldwide (American Psychiatric Association, 2013). A frequent observation is that the prevalence of these disorders is substantially higher in women than in men (McLean et al., 2011; Bandelow and Michaelis, 2015; Craske et al., 2017). While several biological processes are thought to contribute to sexual dimorphism in anxiety disorders, past and recent evidence suggests that sex steroids, as well as their fluctuations in brain systems, are key in mediating these pathological states (Maeng and Milad, 2015; Li and Graham, 2017; Nouri et al., 2022).

Likewise, animal model studies have revealed that females respond to stress and/or fear differently from males (Johnston and File, 1991; Imhof et al., 1993; Scholl et al., 2019; Knight et al., 2021) and dependent on circulating levels of sex steroids (Frye et al., 2000; Marcondes et al., 2001; Gouveia et al., 2004; Lovick, 2014). Therefore, sex steroids may play a role in modulating neurotransmitter systems involved in anxiety-related pathological states. Nevertheless, most studies with animal models are carried out predominantly with males, and their results are generalized to females (Bangasser and Cuarenta, 2021; Shansky and Murphy, 2021). Thus, potential differences between the sexes remain largely unexplored.

Aversive conditioning is one of the most used behavioral paradigms to study fear in rodents (LeDoux, 2014; Fanselow and Wassum, 2015; Izquierdo et al., 2016; Haaker et al., 2019). Regarding sex differences in fear conditioning, conflicting results have been reported. Several studies have shown no sex differences in fear conditioning (Milad et al., 2009a; Cossio et al., 2016; Machado Figueiredo et al., 2019; Carvalho et al., 2021). In contrast, higher conditioned freezing in male compared to female rats (Graham et al., 2009; Daviu et al., 2014; Urien and Bauer, 2022) and increased freezing in females (Baran et al., 2009; Blume et al., 2017) have also been found. When considering the estrous cycle, some studies have indicated low estrogen phases in females to be associated with heightened sensitivity to stress and impaired consolidation of extinction memory (Markus and Zecevic, 1997; Milad et al., 2009a; Zeidan et al., 2011). Others, however, have observed no effects of the estrous cycle on fear conditioning (Cossio et al., 2016; Machado Figueiredo et al., 2019; Carvalho et al., 2021). In fact, we found no sex differences or estrous cycle influence on tone-cued fear conditioning, but female rats displayed lower extinction retention when compared to males in contextual fear conditioning independently of the estrous cycle phase (Reimer et al., 2018).

Several lines of evidence have indicated that dopamine is a key neurotransmitter in fear conditioning (Pezze and Feldon, 2004; De la Mora et al., 2010; Lee et al., 2017; Stubbendorff and Stevenson, 2021). Our group has demonstrated an important involvement of signaling at D2-like dopaminergic receptors in the expression of conditioned fear in rats (De Oliveira et al., 2006, 2009; De Souza Caetano et al., 2013; De Vita et al., 2021). Additionally, the fear response to conditioned stimuli appeared to depend on the activation of the ventral tegmental area–basolateral amygdala dopaminergic connections (De Oliveira et al., 2011, 2013, De Oliveira et al., 2014, 2017; De Souza Caetano et al., 2013). Other studies, on the other hand, have pointed to the importance of D2-like receptors in the nucleus accumbens and infralimbic cortex for fear extinction (Holtzman-Assif et al., 2010; Mueller et al., 2010; Zbukvic et al., 2017). All these studies, however, have overlooked the involvement of dopaminergic mechanisms in conditioned fear in females.

Since there are sex and estrous cycle-dependent fluctuations in dopamine concentration and dopaminergic receptor expression in fear conditioning-relevant brain regions (Xiao and Becker, 1994; Staiti et al., 2011; Locklear et al., 2016; Kokras et al., 2018; Cullity et al., 2019), the present study aimed to explore the involvement of dopamine in conditioned fear in females. For this, we evaluated the effects of blocking D2-like dopaminergic receptors on the expression and extinction of contextual conditioned fear in female rats at different phases of the estrous cycle.

## Materials and Methods

### Animals

Twenty-four male (310–440 g) and 126 female (220–310 g) Wistar rats (8–12 weeks old) from the animal facility of the Federal University of São Carlos (UFSCar) were used. Rats were housed in groups of four per cage (polypropylene boxes, 40 × 33 × 26 cm), under a 12/12 h dark/light cycle (lights on at 07:00 h), at 23 ± 2°C, and given *ad libitum* access to water and rat chow. Experiments were carried out during the light phase of the cycle, between 9:00 and 12:00 h. All procedures were performed in accordance with the National Council for Animal Experimentation Control and were approved by the Committee for Animal Care and Use of the Federal University of São Carlos (Protocol No. 5717160919).

### Assessment of the estrous cycle phase

To assess female rats’ estrous cycle, vaginal smears were taken daily in the morning (09:00 h), starting 10 days before the beginning of the behavioral assays. Vaginal epithelial cell samples were obtained with an inoculation loop, which was initially sterilized in a flame, dipped in 0.9% saline, and then gently inserted into the rat’s vagina. Samples were then smeared onto a microscope glass slide and stained with a staining set (Panótico Rápido, Laborclin, PR, Brazil). After drying, slides were examined under a light microscope. Each phase was identified based on the proportion of epithelial cells, cornified cells, and leukocytes observed in the vaginal smear (Cora et al., 2015; Maeng et al., 2015). Proestrus is characterized by the presence of nucleated cells; in estrus, there is a higher prevalence of cornified, non-nucleated cells; metestrus has equivalent portions of leukocytes, cornified cells, and nucleated epithelial cells; diestrus is characterized by the predominance of leukocytes. The experiments were performed on animals that had completed at least two regular cycles. Rats were grouped by follicular (proestrus/estrus) or luteal (metestrus/diestrus) phases (Reimer et al., 2018), based on the test day of the conditioned fear protocol or the day of the motor evaluation for the catalepsy and open field tests. Sex hormone concentrations were not measured in the present study.

### Drugs

The dopaminergic D2-like antagonist sulpiride (Tocris Bioscience, Bristol, UK) was first mixed in 2% Tween 80 and then dissolved in physiological saline (0.9%) to obtain concentrations of 20 and 40 mg/ml. Physiological saline containing 2% Tween 80 served as vehicle control. Injections were administered in a constant volume of 1 ml/kg, intraperitoneally (i.p.), 15 min before the beginning of the tests. The doses (20 and 40 mg/kg) and injection times were based on previous studies (De Oliveira et al., 2006; Carvalho et al., 2009; De Souza Caetano et al., 2013; De Vita et al., 2021). The investigator was blind to the treatment condition of each rat; drugs were prepared and stored in vials labeled with codes by a different investigator than the one who performed the behavioral experiments and data analysis.

### Contextual fear conditioning

The experimental protocol for contextual fear conditioning was based on Reimer et al. (2018) and De Vita et al. (2021). Contextual fear conditioning procedures took place in standard conditioning chambers (Insight, SP, Brazil). Chamber-A (dimensions: 26 × 20 × 20 cm) was made with white metal walls and ceiling, and a rod floor (5 mm in diameter, spaced 1.5 cm apart) connected to a shock generator (Eltrones, SC, Brazil). Chamber-B (32 × 30 × 30 cm) consisted of stainless steel sides with a clear Plexiglas front, back, and top, with an opaque white polypropylene floor. Conditioning chambers were enclosed in sound-attenuation boxes (66 × 43 × 45 cm; Grason-Stadler, MN, USA). Chamber-A was equipped with a fan providing constant background noise. Alcohol (20%) was used as a mild scent in Chamber-A; acetic acid (2%) odor was added to Chamber-B. Animals’ behavior was recorded with digital video cameras positioned in front of the chambers. Videos were evaluated by a blind rater after the tests were completed. Freezing behavior was scored as an index of the contextual fear conditioning and it was operationally defined as the total absence of movements, except those required for respiration, for at least 6 s per episode.

### Catalepsy bar test

The apparatus and testing procedures used for the catalepsy test have been described in detail elsewhere (Colombo et al., 2013; Barroca et al., 2019; Waku et al., 2022). Briefly, a horizontal acrylic bar (30 cm in length and 1 cm in diameter) was positioned 8 cm above the floor of a standard polypropylene box (40 × 33 × 26 cm). The animal’s forepaws were carefully positioned on the bar, while their hind paws remained on the ground. The latency to step down from the horizontal bar was measured 15 and 45 min after drug administration.

### Open-field test

During the interval between the catalepsy bar tests, the animals underwent the open-field test. The experimental protocol for the open-field test was based on Reimer et al. (2012) and De Vita et al. (2021). Locomotor activity was assessed in a circular arena consisting of a transparent acrylic enclosure (60 cm in diameter, 50 cm in height, floor divided into 12 sections). Rats were placed in the middle of the arena and left for a 15-min period of free exploration. The session was recorded by a camera positioned above the open-field. At the end of the test, the total number of crossings (number of floor sections crossed) and rearings (standing with forepaws raised in the middle of the arena or against the walls) were analyzed.

### Experiment 1: contextual fear as a function of sex and estrous cycle in female rats

**Figure 1:**
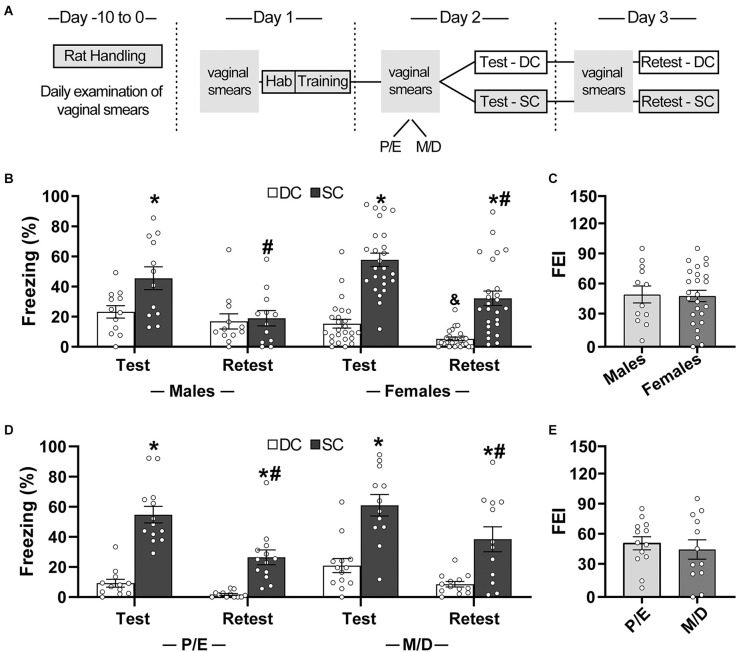
Expression (test) and extinction (retest) of contextual conditioned fear did not differ between male and female rats. **(A)** Timeline of the experimental procedure. **(B,D)** Mean percentage of freezing during test and retest sessions (time spent freezing during session/session duration *100) for different-context (DC) and same-context (SC) groups. **(C,E)** Fear extinction index (FEI; % freezing exhibited during the final block of the training session − % freezing exhibited during retest session). *p < 0.05: different from the DC group in the same session; ^#^*p* < 0.05: different from the same group during the test session; ^&^*p* < 0.05: different from the male DC group at retest. Males: *n* = 12 for DC and SC; Females proestrus/estrus (P/E): *n* = 12 for DC and 13 for SC; Females metestrus/diestrus (M/D): *n* = 13 for DC and 12 for SC.

Initially, we aimed at validating our protocol while exploring the influence of sex and estrous cycle on contextual fear conditioning (Figure 1A). For that, rats were divided into two groups: Same Context and Different Context groups. Both groups underwent training in Chamber-A. During the training session, animals were placed individually into the experimental chamber and, after a habituation period of 5 min, 10 1-s unsignaled footshocks (0.6 mA, inter-trial interval 30–90 s) were presented. Each animal was removed 2 min after the last footshock and returned to its home cage. The training session lasted approximately 15 min. After 24 h, animals were submitted to the test session, without shock presentation; the Same Context group was reexposed to Chamber-A while the Different Context group was placed into Chamber-B. Animals remained in the behavioral chambers for 10 min. The retest session was performed 24 h after the test session. Animals were again re-exposed to the same chambers used during the test session for a total of 10 min.

### Experiment 2: involvement of D2-like dopaminergic receptors in the expression and extinction of conditioned fear in female rats

**Figure 2:**
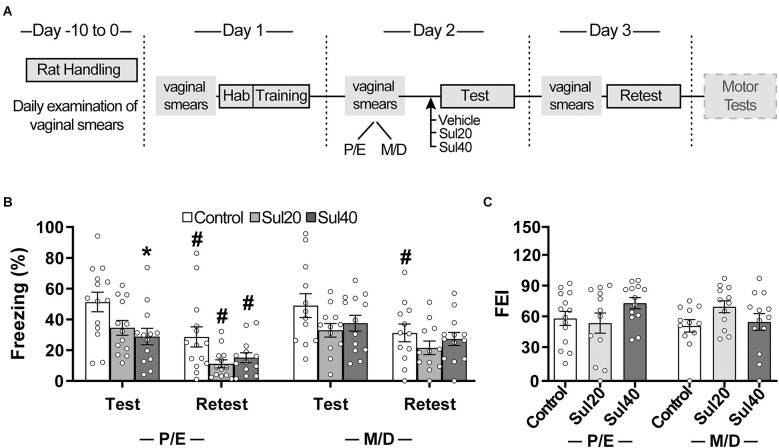
Sulpiride decreased the expression of conditioned freezing in female rats. **(A)** Timeline of the experimental procedure. **(B)** Effects of sulpiride 20 and 40 mg/kg on the expression (test) and extinction (retest) of contextual conditioned freezing in female rats in proestrus/estrus (P/E) and metestrus/diestrus (M/D). Mean percentage of freezing during test and retest sessions (time spent freezing during session/session duration*100). **(C)** Fear extinction index (FEI; % freezing exhibited during the final block of the training session − % freezing exhibited during retest session). *p < 0.05: different from the control group in the same session; ^#^*p* < 0.05: different from the same group during the test session. Females P/E: *n* = 14 for Control, 12 for Sul20, 13 for Sul40; Females M/D: *n* = 12 for Control, 12 for Sul20, 13 for Sul40.

**Figure 3:**
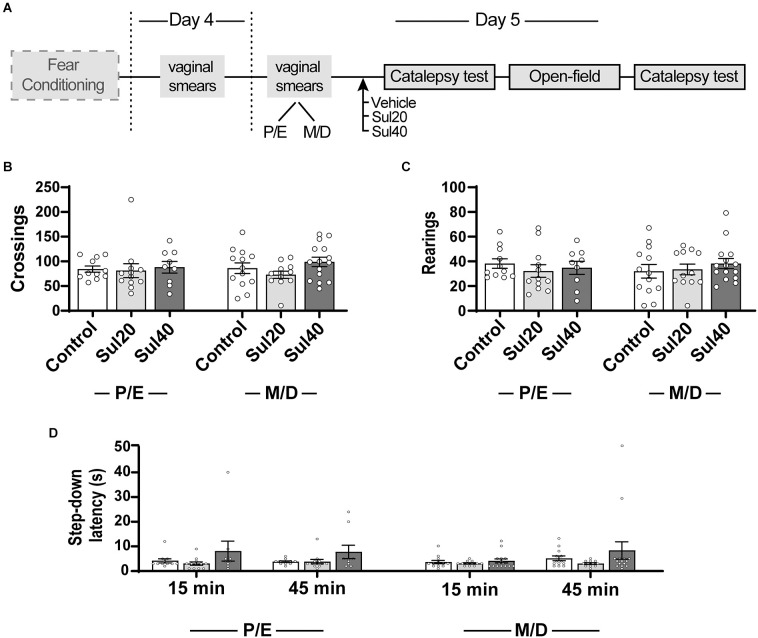
Sulpiride 20 and 40 mg/kg did not affect motor performance in the open-field and catalepsy tests in female rats in proestrus/estrus (P/E) and metestrus/diestrus (M/D). **(A)** Timeline of the experimental procedure. **(B)** The total number of crossings in the open-field test. **(C)** The total number of rearings in the open-field test. **(D)** Latency to step-down in the catalepsy test 15 and 45 min after treatments. Females P/E: *n* = 11 for Control, 12 for Sul20, 9 for Sul40; Females M/D: = 13 for Control, 12 for Sul20, 15 for Sul40.

Experiment 2’s fear conditioning protocol followed the same procedures described for Experiment 1, in which rats were submitted to three consecutive sessions (training, test, and retest) spaced by 24 h each, but no different context group was used this time (Figure 2A). Briefly, animals were submitted to training in Chamber-A for aversive conditioning. On the second day (test session), animals received i.p. injections of vehicle or drug treatment and, after 15 min, they were reexposed to the Chamber-A for 10 min. Twenty-four hours after the test, rats were once again placed in Chamber-A for 10 min (retest session), for evaluation of extinction recall. Potential nonspecific effects of sulpiride on motor behavior were evaluated in the same animals. For this, 48 h after the end of the fear conditioning retest session, animals were subjected to the open-field and catalepsy bar tests (Figure 3A).

### Analysis of results

All analyses were conducted using R version 4.0.5 (R Core Team, 2020) and Rstudio version 1.4.1717 (RStudio Team, 2020). Prior to model fitting, we used the fitdistrplus package version 1.1-8 (Delignette-Muller and Dutang, 2015) to assess the distribution of each response variable by plotting a Cullen-Frey diagram with 1,000 bootstraps. Additionally, using the fitdistrplus’s fitdist plot function, we drew four classical goodness-of-fit plots (i.e., density plot, CDF plot, Q-Q plot, and P-P) to compare the fit of other candidate distributions that are often used in fear conditioning paradigm and motor function assessment analyses. Based on this, for the fear conditioning experiments, beta regression analysis was selected because it was a more appropriate fit to the data distribution than the commonly used repeated measures ANOVA. Behavioral scores were rescaled to between 0 and 1 using a min-max normalization. Generalized linear mixed models (GLMMs) with logit link function and beta distribution were used. For Experiment 1, rats’ identities were used as random factors while Group (Same-Context and Different-Context), Session (Test and Retest), and Sex (Male and Female) or Phase (Proestrus/Estrus and Metestrus/Diestrus) were used as fixed factors. For Experiment 2, rats’ identities were used as random factors, Phase (Proestrus/Estrus and Metestrus/Diestrus), Session (Test and Retest), and Treatment (Saline, Sulpiride-20, Sulpiride-40) were used as fixed factors. For the assessment of motor function, generalized linear models (GLMs) with log link function and negative binomial distribution were used to evaluate the frequency of crossings and rearings. Phase (Proestrus/Estrus and Metestrus/Diestrus) and Treatment (Saline, Sulpiride-20, Sulpiride-40) were used as fixed factors. GLMMs with logit link function and beta distributions were used to evaluate step-down latency in the catalepsy test. Rats’ identities were used as random factors, Phase (Proestrus/Estrus and Metestrus/Diestrus), Time (15- and 45-min), and Treatment (Saline, Sulpiride-20, Sulpiride-40) were used as fixed factors. Model fitting was performed with the glmmTMB package version 1.1.3 (Brooks et al., 2017). Likelihood ratio tests were conducted using car package version 3.1-0 (Fox and Weisberg, 2019). Planned *a priori* pairwise comparisons were tested using the emmeans package 1.8.1-1 (Lenth, 2021) and Holm’s sequential Bonferroni procedure was used to adjust the significance level due to the multiple comparisons.

## Results

### Experiment 1: contextual fear as a function of sex and estrous cycle in female rats

Male and female rats submitted to the training and testing procedures in the same cage (Same Context) spent more time freezing than rats submitted to the test session in a cage different from the one used for training (Different Context; Figures 1B,C; Supplementary Figure 1). An analysis of variance based on mixed beta regression indicated statistically significant effects for Group (Different × Same-Context: χ^2^ = 35.57, *p* < 0.05), Session (Test × Retest: χ^2^ = 19.27, *p* < 0.05), and Sex (Male × Female: χ^2^ = 7.58, *p* < 0.05). Pairwise comparisons using t-tests, corrected with Holm’s sequential Bonferroni procedure, indicated that the same-context group froze more than the different-context at the test session in both males (*t* = −2.66, *p* < 0.05) and females (*t* = −7.52, *p* < 0.05). During the retest, the same-context continued to freeze more than the different-context group in females (*t* = −5.96, *p* < 0.05), but not in males (*t* = 0.17, *p* > 0.05). Decreased freezing during retest was observed for same-context males (*t* = −6.11, *p* < 0.05) and females (*t* = −8.07, *p* < 0.05). Males and females did not differ in the freezing response during the test session either for the same-context (*t* = −1.86, *p* > 0.05) or different-context groups (*t* = 1.27, *p* > 0.05). During the retest, females of the different-context group froze less than males (*t* = 2.75, *p* < 0.05), but males and females of the same-context groups did not differ from each other (*t* = −2.43, *p* > 0.05). No significant difference between sexes was observed for the fear extinction index (FEI; Male × Female: χ^2^ = 0.22, *p* > 0.05).

There was no difference in freezing between phases of the estrous cycle for the same-context or different context groups (Figures 1D,E). An analysis of variance based on mixed beta regression indicated statistically significant effects for Group (Different × Same-Context: χ^2^ = 17.10, *p* < 0.05), Session (Test × Retest: χ^2^ = 10.76, *p* < 0.05), and Cycle-Phase (Proestrus/Estrus × Metestrus/Diestrus: χ^2^ = 4.57, *p* < 0.05). Pairwise comparisons using t-tests, corrected with Holm’s sequential Bonferroni procedure, indicated that the same-context group froze more than the different-context at the test and retest sessions in both proestrus/estrus (test: *t* = −6.12, *p* < 0.05; retest: *t* = −4.87, *p* < 0.05) and metestrus/diestrus groups (test: *t* = −5.23, *p* < 0.05; retest: *t* = −4.14, *p* < 0.05). Decreased freezing during retest was observed for same-context proestrus/estrus (*t* = −6.28, *p* < 0.05) and metestrus/diestrus females (*t* = −5.46, *p* < 0.05). Proestrus/estrus and metestrus/diestrus females did not differ in the freezing response during the test or retest sessions either for the same-context (test: *t* = 0.95, *p* > 0.05; retest: *t* = 1.26, *p* > 0.05) or different-context groups (test: *t* = 1.97, *p* > 0.05; retest: *t* = 2.14, *p* > 0.05). No significant difference between phases of the estrous cycle was observed for the fear extinction index (FEI; Proestrus/Estrus × Metestrus/Diestrus: χ^2^ = 0.20, *p* > 0.05).

### Experiment 2: involvement of D2-like dopaminergic receptors in the expression and extinction of conditioned fear in female rats

Sulpiride decreased contextual conditioned freezing response in female rats (Figure 2; Supplementary Figure 2). An analysis of variance based on mixed beta regression indicated statistically significant effects for Treatment (Saline × Sulpiride-20 × Sulpiride-40: χ^2^ = 13.30, *p* < 0.05), Session (Test × Retest: χ^2^ = 84.61, *p* < 0.05), but not for Cycle-Phase (Proestrus/Estrus × Metestrus/Diestrus: χ^2^ = 2.97, *p* > 0.05). Pairwise comparisons using t-tests, corrected with Holm’s sequential Bonferroni procedure, indicated decreased freezing response for sul20 and sul40 compared to the control group during the test session (*t* = 3.42, *p* < 0.05 and *t* = 3.50, *p* < 0.05, respectively). Freezing response did not differ for sul20 and sul40 compared to the control group during the retest session (*t* = 2.65, *p* > 0.05 and *t* = 1.62, *p* > 0.05, respectively). Pairwise comparisons also indicated decreased freezing during retest for control (*t* = −7.00, *p* < 0.05), sul20 (*t* = −5.29, *p* < 0.05), and sul40 (*t* = −3.91, *p* < 0.05) compared to the test session.

When considering the estrous cycle phases, pairwise comparisons using t-tests, corrected with Holm’s sequential Bonferroni procedure, indicated decreased freezing response for sul40 in proestrus/estrus compared to the control group during the test session (*t* = 2.92, *p* < 0.05), but not for sul20 in proestrus/estrus (*t* = 2.32, *p* > 0.05) or sul20 and sul40 in metestrus/diestrus (*t* = 2.51, *p* > 0.05 and *t* = 2.04, *p* > 0.05, respectively). Freezing response did not differ for sul20 and sul40 compared to the control group during the retest session for proestrus/estrus (sul20: *t* = 2.66, *p* > 0.05; sul40: *t* = 1.95, *p* > 0.05) or metestrus/diestrus (sul20: *t* = 1.08, *p* > 0.05; sul40: *t* = 0.33, *p* > 0.05). Pairwise comparisons also indicated decreased freezing during retest compared to the test session for control in proestrus/estrus (*t* = −5.10, *p* < 0.05) and metestrus/diestrus (*t* = −4.83, *p* < 0.05), and for sul20 and sul40 in proestrus/estrus (*t* = −4.82, *p* < 0.05 and *t* = −3.19, *p* < 0.05, respectively), but not for sul20 and sul40 in metestrus/diestrus (*t* = −2.55, *p* > 0.05 and *t* = −2.30, *p* > 0.05, respectively). No significant differences were observed for the fear extinction index between sul20 or sul40 and the control group during proestrus/estrus (*t* = 0.46, *p* > 0.05; *t* = −1.40, *p* > 0.05, respectively) or metestrus/diestrus (*t* = −2.48, *p* > 0.05; *t* = −1.15, *p* > 0.05, respectively).

Sulpiride did not affect motor performance evaluated with the open-field and catalepsy tests (Figure 3). For the open-field test, analysis of variance based on mixed negative binomial regression revealed no statistically significant for crossings (Treatment—Saline × Sulpiride-20 × Sulpiride-40: χ^2^ = 3.15, *p* > 0.05; Cycle-Phase—Proestrus/Estrus × Metestrus/Diestrus: χ^2^ = 0.08, *p* > 0.05) or rearings (Treatment—Saline × Sulpiride-20 × Sulpiride-40: χ^2^ = 0.78, *p* > 0.05; Cycle-Phase—Proestrus/Estrus × Metestrus/Diestrus: χ^2^ = 0.34, *p* > 0.05). Pairwise comparisons using t-tests, corrected with Holm’s sequential Bonferroni procedure, indicated no differences for crossings between sul20 or sul40 and the control group during proestrus/estrus (*t* = 0.66, *p* > 0.05; *t* = 0.04, *p* > 0.05, respectively) or metestrus/diestrus (*t* = 0.84, *p* > 0.05; *t* = −1.18, *p* > 0.05, respectively). Pairwise comparisons also indicated no differences for rearings between sul20 or sul40 and the control group during proestrus/estrus (*t* = 1.17, *p* > 0.05; *t* = 0.76, *p* > 0.05, respectively) or metestrus/diestrus (*t* = −0.62, *p* > 0.05; *t* = −1.64, *p* > 0.05, respectively). For the catalepsy test, an analysis of variance based on mixed beta regression indicated statistically significant effects for Time (15 × 45 min: χ^2^ = 7.80, *p* < 0.05), but not for Treatment (Saline × Sulpiride-20 × Sulpiride-40: χ^2^ = 1.30, *p* > 0.05) or Cycle-Phase (Proestrus/Estrus × Metestrus/Diestrus: χ^2^ = 0.23, *p* > 0.05). Pairwise comparisons using t-tests, corrected with Holm’s sequential Bonferroni procedure, indicated no differences between sul20 and the control group during proestrus/estrus (15-min: *t* = 1.16, *p* > 0.05; 45-min: *t* = 0.33, *p* > 0.05) or metestrus/diestrus (15-min: *t* = 0.16, *p* > 0.05; 45-min: *t* = 1.17, *p* > 0.05). Pairwise comparisons also indicated no differences between sul40 and the control group during proestrus/estrus (15-min: *t* = −1.09, *p* > 0.05; 45-min: *t* = −1.05, *p* > 0.05) or metestrus/diestrus (15-min: *t* = 0.72, *p* > 0.05; 45-min: *t* = −1.56, *p* > 0.05).

## Discussion

The present study aimed to evaluate the involvement of dopamine D2-like receptors on contextual fear conditioning in female rats. Blockade of D2-like receptors significantly decreased the expression of freezing response in female rats. Moreover, females in proestrus/estrus phases were more sensitive to the effects of sulpiride than females in metestrus/diestrus. These findings suggest a functional interplay between the estrous cycle phase and dopaminergic neurotransmission in fear conditioning. The effect of blocking D2-like receptors was specific to the fear response, with no motor impairment observed during the different phases of the estrous cycle.

The protocol used for acquisition, expression, and extinction of contextual conditioned fear was effective for both males and females. This result is in line with other studies from our group obtained using a similar protocol in male rats only (De Souza Caetano et al., 2013; De Vita et al., 2021) and extends the validity of our protocol to female rats. We did not verify differences between the sexes or estrous cycle phases for the expression and extinction of conditioned freezing, although same-context females continue to freeze more in the retest than different-context females. No differences were observed in the fear extinction index. Literature has been reporting mixed results regarding sex/estrous cycle differences in fear conditioning paradigms. Previous studies have reported either higher freezing expression in male rats (Graham et al., 2009; Daviu et al., 2014; Urien and Bauer, 2022), female rats (Baran et al., 2009; Blume et al., 2017), or no difference (Cossio et al., 2016; Zhao et al., 2018; Machado Figueiredo et al., 2019; Carvalho et al., 2021). There may be several explanations for this, particularly regarding differences in methods, subject strain, determination of gonadal hormonal status, and fear conditioning protocol (e.g., cued or contextual CS) used in those studies.

These potential inconsistencies are raising increasingly important questions regarding the participation of different neurotransmitter/neuromodulator/neurosteroid systems influencing aversive learning and memory. Several pieces of evidence point to different mechanisms by which fear processing could diverge in male and female subjects. Neuronal activity in the basolateral amygdala shows faster neuron firing rates and greater excitatory synaptic input in females (Blume et al., 2017). Different activation patterns in the hippocampus, amygdala, and bed nucleus of the stria terminalis during fear conditioning paradigms are found between male and female rats (Maren et al., 1994; Blume et al., 2017; Keiser et al., 2017; Urien and Bauer, 2022). Also, the distinct participation of neurotransmitter systems regulating fear response, particularly endocannabinoid (Morena et al., 2021), serotonergic and dopaminergic systems (Mitsushima et al., 2006; Rey et al., 2014) have been described.

Previous works from our group have demonstrated that the blockade of dopamine D2-like receptors with sulpiride impairs fear expression in male rats (De Oliveira et al., 2011, 2013, De Oliveira et al., 2017; De Souza Caetano et al., 2013; De Vita et al., 2021). Here, sulpiride significantly reduced freezing response in female rats in a similar fashion. Some studies reported that systemically administration of the D2-like antagonists’ haloperidol and raclopride to males have, however, increased freezing behavior (Holtzman-Assif et al., 2010; Mueller et al., 2010). These studies did not assess the motor side effects of these drugs or could not dissociate the effects on freezing from impaired motor behavior. Sulpiride’s effect on the freezing response observed in females in the present study cannot be attributed to nonspecific motor effects, as it neither affected animals’ performance in catalepsy nor open-field tests. Likewise, we did not observe motor effects in males using similar doses (De Vita et al., 2021). Sulpiride’s effect on conditioned fear without adversely affecting motor function suggests a stronger action of sulpiride on the mesolimbic than in the nigrostriatal dopaminergic pathway. In this direction, the atypical neuroleptic amisulpride, which bears a close chemical and clinical resemblance to sulpiride, showed greater selectivity for limbic than striatal dopaminergic projections (Schoemaker et al., 1997).

In addition to demonstrating that sulpiride significantly reduced freezing expression in female rats, we found that the females’ responsiveness to sulpiride appears to be dependent on the estrous cycle phase. A significant reduction in the expression of contextual conditioned freezing behavior after sulpiride administration was observed in females in proestrus/estrus, but not in metestrus/diestrus. Sulpiride’s lack of effect in metestrus/diestrus suggests that the brain’s hormonal profile during proestrus/estrus favors the action of the D2-like antagonist. We also observed estrous cycle-dependent effects on contextual fear in a previous study in which deficits induced by meta-chlorophenylpiperazine (mCPP)—a serotonergic agonist—prevailed in metestrus/diestrus but not proestrus/estrus or in males, suggesting that fluctuations in sex hormones may also influence the action of serotonergic drugs (Reimer et al., 2018).

Hormonal fluctuations throughout the cycle could lead to differences in the response to pharmacological manipulations. It is possible, for example, that susceptibility to the effects of sulpiride may result from differences in dopamine availability or the expression of D2-like receptors (Xiao and Becker, 1994; Thompson and Moss, 1997; Le Saux et al., 2006; Diekhof and Ratnayake, 2016; Petersen et al., 2021). Our findings suggest that D2-like receptors play an important role in the expression of conditioned fear in females in proestrus/estrus in a manner similar to that observed in previous studies with males (De Oliveira et al., 2006, 2013; De Souza Caetano et al., 2013; De Vita et al., 2021). The fact that female rats tested during higher hormone levels respond to the pharmacological manipulation similarly to males might be attributed to the role of estradiol, in both sexes (Lebron-Milad and Milad, 2012; Graham and Milad, 2013, 2014). Although it seems counterintuitive given that estradiol levels in male rats would be comparable to those found in low-estradiol females, many effects of testosterone actually seem to be mediated by estradiol (Purves-Tyson et al., 2007; Hammes and Levin, 2019; Zuloaga et al., 2020). Estradiol in males can be synthesized via aromatization of circulating testosterone as well as from testosterone produced de novo by neurons and glia (Gillies and McArthur, 2010). This would explain why sex differences arose in females tested in metestrus/diestrus. It is important to highlight, however, that hormones were not measured in the present study.

In male rats, besides recently confirming the involvement of D2-like receptor-mediated mechanisms in the expression of conditioned fear, our previous results have suggested that D2-like receptors are not involved in conditioned freezing extinction (De Vita et al., 2021). In the present study, sulpiride administered to females in proestrus/estrus during the test session did not affect the freezing behavior exhibited 24 h later, during the retest session. When administered to metestrus/diestrus females during the test session, sulpiride did not alter freezing in the retest when compared to the vehicle control group, but it prevented the expected decrease in freezing in the retest compared to the test session. The analysis of the fear extinction index, however, did not indicate an effect of sulpiride on extinction. The effects of systemically administered drugs acting at D2-like receptors on contextual fear extinction are, in fact, not well understood (Stubbendorff and Stevenson, 2021). In studies using cued-fear conditioning, systemic haloperidol and raclopride administration have been shown to decrease extinction recall (Holtzman-Assif et al., 2010; Mueller et al., 2010). Systemic injections of sulpiride in male mice have been shown to facilitate the extinction of cued fear conditioning only in a protocol that did not promote extinction (Ponnusamy et al., 2005). Altogether, data point to a modulatory rather than an essential role for D2-like receptors in fear extinction.

Although our results implicate dopaminergic D2-like receptors in conditioned fear in females, the role of specific dopaminergic areas remains to be demonstrated. The present results agree with those reported by others showing decreased conditioned freezing in males with sulpiride locally administered to the amygdala and prefrontal cortex (De Oliveira et al., 2011, 2017; De Souza Caetano et al., 2013; Dadkhah et al., 2021). In addition, exposure to aversive conditioned stimuli has been shown to increase dopamine levels in the basolateral amygdala, nucleus accumbens, and prefrontal cortex in male rats (Pezze et al., 2001; Matsumoto et al., 2005; Martinez et al., 2008; De Oliveira et al., 2011, 2013, De Oliveira et al., 2014). It will be important to determine whether the effects of sulpiride in females are also observed after pharmacological manipulations of dopaminergic neurotransmission in regions of the mesocorticolimbic system. Ongoing work in our laboratory is assessing the effects of intra-basolateral amygdala dopaminergic compounds in female rats submitted to contextual fear conditioning. In addition to the role of dopamine, we have previously shown that contextual conditioned freezing was inhibited by GABA-A receptor agonist muscimol injections into the basolateral amygdala in males (Martinez et al., 2006). Future studies should explore the GABA-dopamine relationship in contextual conditioned fear in males and females at different phases of the estrous cycle.

The study of fear conditioning in rodents has clinical relevance, contributing to a better understanding of the pathophysiology and treatment of human mental disorders such as post-traumatic stress disorder and obsessive-compulsive disorder (Milad et al., 2009b; 2013; Indovina et al., 2011; Izquierdo et al., 2016; Dougherty et al., 2018; Sevenster et al., 2018; Bienvenu et al., 2021; Cooper and Dunsmoor, 2021). The neglect of using female subjects in preclinical neuroscience research, however, contributes to an incomplete understanding of the pathophysiology and treatment of anxiety-related mental disorders in women (Beery and Zucker, 2011; Shansky and Murphy, 2021). The present results reinforce the importance of dopaminergic mechanisms in fear conditioning by demonstrating the involvement of D2-like receptors during the contextual conditioned freezing response in female rats. The present results also indicate that D2-like receptors in females are mainly involved in the expression of fear states during proestrus/estrus rather than in metestrus/diestrus.

## Data Availability Statement

The raw data supporting the conclusions of this article will be made available by the authors, without undue reservation.

## Ethics Statement

The animal study was reviewed and approved by Committee for Animal Care and Use of the Federal University of São Carlos (protocol no. 5717160919).

## Author Contributions

AO designed the study and supervised the project. CA performed the research. AR performed the analysis and compiled the figures. AO, AR, and CA wrote the manuscript. All authors contributed to the article and approved the submitted version.

## Funding

This study was supported by the Brazilian National Council for Scientific and Technological Development (CNPq Proc. No. 401032/2016-7), the Coordenação de Aperfeiçoamento de Pessoal de Nível Superior–Brasil (CAPES Finance Code 001), and the São Paulo Research Foundation (FAPESP Proc. No. 2016/04620-1). CA was supported by a Master’s fellowship (Proc. No. 2019/20274-4) and holds a Doctoral fellowship (Proc. No. 2021/04949-1) both from FAPESP. AR holds a Postdoctoral Young Talents fellowship from CAPES(88887.576069/2020-00).

## Conflict of Interest

The authors declare that the research was conducted in the absence of any commercial or financial relationships that could be construed as a potential conflict of interest.

## Publisher’s Note

All claims expressed in this article are solely those of the authors and do not necessarily represent those of their affiliated organizations, or those of the publisher, the editors and the reviewers. Any product that may be evaluated in this article, or claim that may be made by its manufacturer, is not guaranteed or endorsed by the publisher.

## Supplementary Material

The Supplementary Material for this article can be found online at: https://www.frontiersin.org/articles/10.3389/fnbeh.2022.1033649/full#supplementary-material.
