# Supplementary material for: Involvement of D2-like dopaminergic receptors in contextual fear conditioning in female rats: influence of estrous cycle
Source: Front Behav Neurosci. 2022 Nov 28;16:1033649. doi: 10.3389/fnbeh.2022.1033649 (PMC9742248; doi:10.3389/fnbeh.2022.1033649)
Supplement: Supplementary file 1 [file Data_Sheet_1.docx]

# **Supplementary Material**


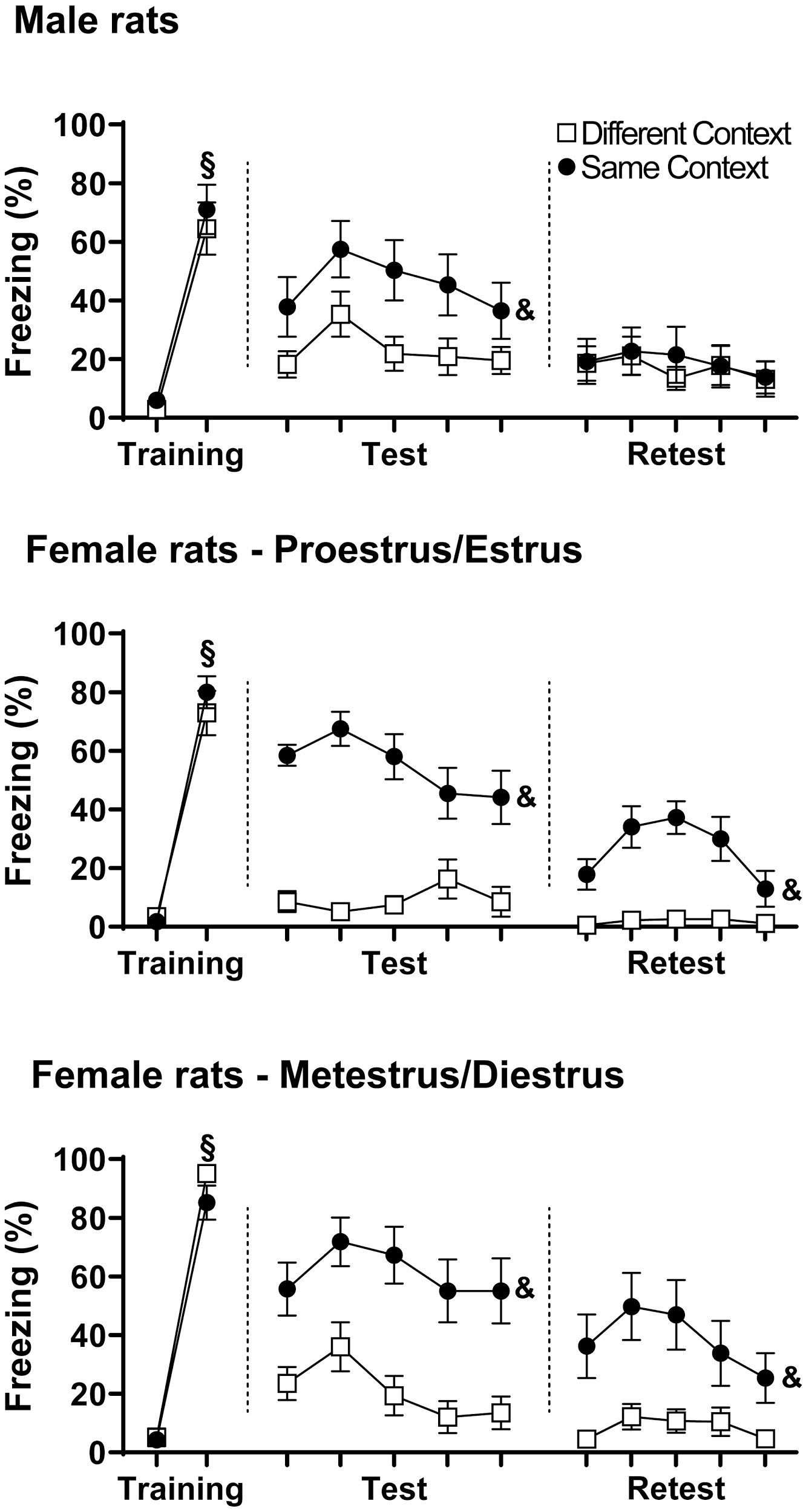


**Supplementary Figure 1.** Contextual conditioned freezing in male rats and female rats in proestrus/estrus and metestrus/diestrus. Mean percentage of freezing (blocks of 2 min) for training (initial and final blocks), test and retest sessions (time spent freezing during the block/block duration*100). §p < 0.05: different from the initial phase of the training session; &p < 0.05: different from the different context group (DC). Males: n = 12 for DC and SC; Females P/E: n = 12 for DC and 13 for SC; Females M/D: n = 13 for DC and 12 for SC.


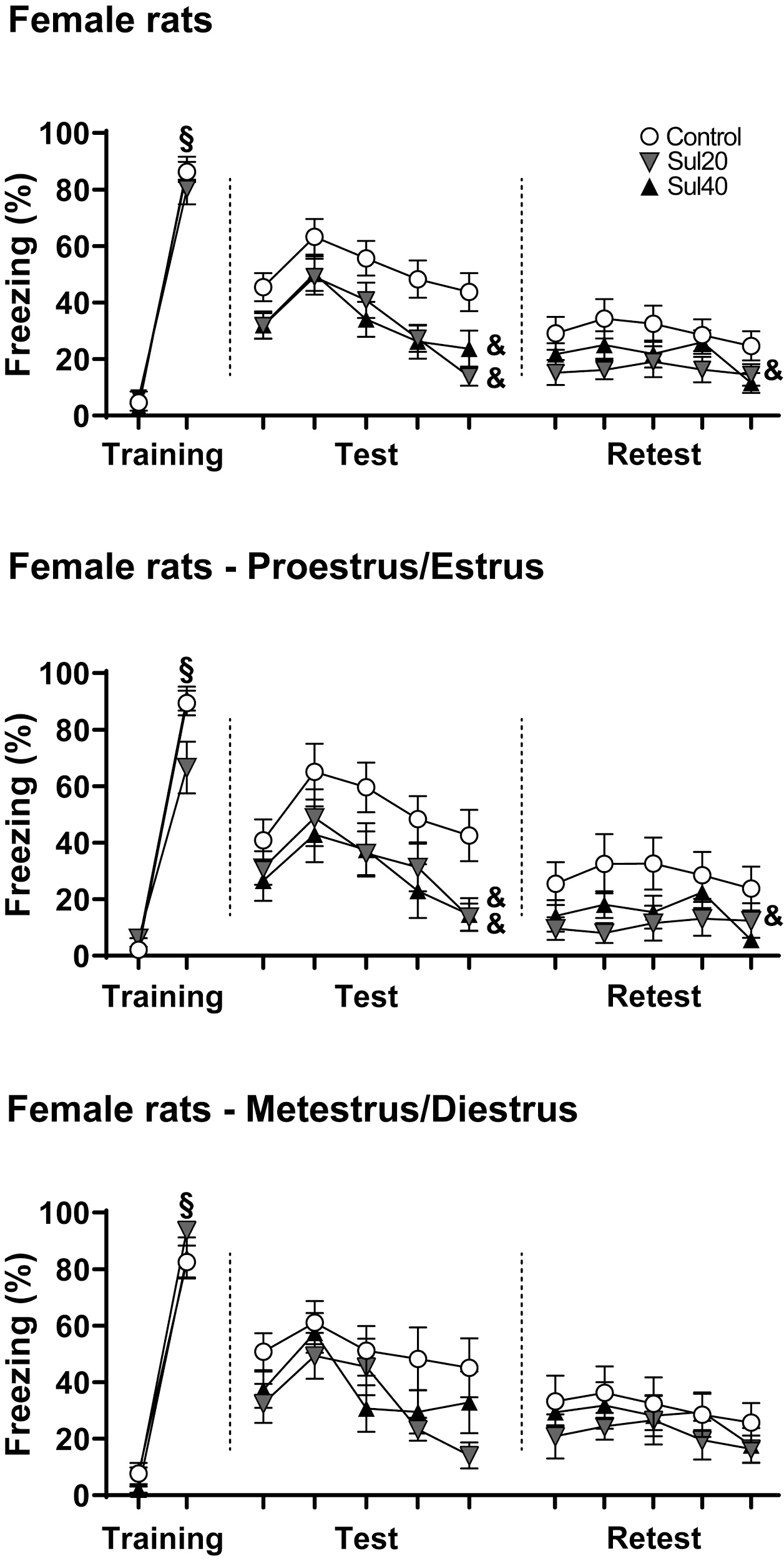


**Supplementary Figure 2.** Effects of sulpiride 20 and 40 mg/kg on the expression (test) and extinction (retest) of contextual conditioned freezing in female rats. Mean percentage of freezing (blocks of 2 min) for training (initial and final blocks), test and retest sessions (time spent freezing during the block/block duration*100). §p < 0.05: different from the initial phase of the training session; &p < 0.05: different from the control group. Females: n = 26 for Control, 24 for Sul20, 26 for Sul40; Females P/E: n = 14 for Control, 12 for Sul20, 13 for Sul40; Females M/D:  n = 12 for Control, 12 for Sul20, 13 for Sul40.
